# COVID-19 Management Missteps

**DOI:** 10.7759/cureus.23059

**Published:** 2022-03-11

**Authors:** F Brian Boudi, Sabin Patel, Kajal Patel, Kajal Parikh, Neha Patel, Max Boudi, Samir Patel, Himanshu Patel

**Affiliations:** 1 Cardiology, University of Arizona College of Medicine, Phoenix, USA; 2 Biology, Baylor University, Waco, USA; 3 Biology, Emory University, Atlanta, USA; 4 Biological Sciences, Rutgers University, New Brunswick, USA; 5 Psychology, University of Alabama at Birmingham, Birmingham, USA; 6 Education, Arizona State University, Phoenix, USA; 7 Biomedical Engineering, Arizona State University, Phoenix, USA; 8 Psychiatry, University of Arizona College of Medicine, Phoenix, USA

**Keywords:** covid-19 vaccination, epidemiology and biostatistics, public health and safety, viral infection, covid-19

## Abstract

In December 2019, the first case of a novel coronavirus infectious disease, coronavirus disease 2019 (COVID-19), was identified in the province of Wuhan, China. Since the initial identification on March 11, 2020, by the World Health Organization (WHO), COVID-19 had rapidly spread all over the world, leading to the declaration of COVID-19 as a pandemic. In response to the exponential trend of reported confirmed cases, national governments worked quickly to devise plans to combat the spread and to soften the consequences which were to follow. Two primary approaches included limiting the spread of the virus and increasing hospital capacity. The implementation of these strategies, however, varied greatly among different governments and their respective populations.

Countries developed similar guidelines in response to COVID-19, but with a variation. Many of these guidelines were similar in that they fell under the same general topics such as the use of facial masks, social distancing, and online learning. The effect of COVID-19 on public health was more reliant on the implementation of these recommendations rather than the recommendations themselves.

The medical therapies used to treat the widespread COVID-19 disease are flourishing and evolving rapidly. Ongoing research shows that the spectrum of treatment for COVID-19 varies from pharmacological and non-pharmacological therapeutic interventions. Some of the treatments that are being used in clinical practice include supportive care, antiviral drugs, immunomodulatory agents, convalescent plasma transfusion, and monoclonal antibody treatments. In addition, the most promising approach thus far is the COVID-19 vaccine developed by Pfizer-BioNTech, Moderna, and most recently Johnson & Johnson. Overall, as various treatment approaches are being explored and administered to people globally, it is important to acknowledge that there is currently no definite cure or any evidence-based treatment for COVID-19.

COVID-19 infections caused by severe acute respiratory syndrome coronavirus 2 (SARS-CoV-2) have brought devastating consequences to the lives of millions of people through their health effects and the failure of global initiatives to contain it. A review of many missteps that potentially could have altered the landscape for this virus to affect the lives of many is discussed with hope for a better approach going forward.

## Introduction and background

Variations of safety recommendations from the start

Similar to other countries, the United States knew that the public needed to follow general guidelines for preventing the spread of the virus. However, unlike the U.S., the preexisting culture of wearing masks within East Asian countries from the experience of past epidemics in addition to the general awareness of public health awareness helped its citizens to quickly begin mask adoption as a method of precaution [1]. In comparison, the U.S. Centers for Disease Control and Prevention (CDC) did not recommend it as quickly as these countries. In fact, they only started to recommend indoor use of masks in December 2020 [2]. For more than five weeks after the virus was first documented in the U.S., the CDC considered public mask-wearing to be unnecessary. However, it was only in April that the CDC reversed its stance and advised people to wear cloth masks when in public spaces. Another measure for prevention when traveling to public places required human interaction outside of their households but interestingly the recommended distance varied in different countries [3]. According to the World Economic Forum, “China, Denmark, and France recommend social distancing of one meter; Australia, Germany, and Italy recommend 1.5 meters, and the U.S. recommends six feet, or 1.8 meters,” showing how diverse the safety recommendations were in different countries [4]. 

Treatment approaches

COVID-19 is a respiratory droplet-mediated infectious disease caused by the new SARS-CoV-2 virus. SARS-CoV-2 has infected over a million people across the world, classifying COVID-19 as a worldwide pandemic. Current approaches to COVID-19 therapies have their own mechanisms to treat patients infected by the virus. The first line of treatment started with supportive therapy, which is implemented to help relieve the symptoms of COVID-19 and help to prevent further degradation of health [5]. More pharmacological approaches include antivirals which work to prevent the virus from replicating, and immune modulators, which act to help the immune system fight the virus or stop it from overreacting dangerously [6]. Antivirals and immune modulators play a key role in fighting viruses and preventing future infection by the same virus. In efforts to combat the spread of coronavirus, researchers have developed two types of immunotherapy treatments that utilize antibodies. Convalescent plasma transfusion utilizes the blood of donors who have recovered from COVID-19, which may contain antibodies that suppress the virus, and transfuses it into sick patients. On the other hand, monoclonal antibody therapy is done by giving a patient an infusion of laboratory-made antibodies that target the spike protein that the virus uses to enter host cells [7]. In addition to conducting research for these treatment approaches, the government has also been working towards the creation and distribution of the COVID-19 vaccines in the hope of establishing a long-term treatment approach and a method of prevention for the spread of this disease. 

The economic impact of the shutdown

One specific measure, lockdown, has generated much debate concerning the potential inefficiency of the policy. The inefficiency stems from the resistance on the part of policymakers and stakeholders as lockdowns produce a high economic price, distinguished by the necessity to temporarily cease numerous productive activities. Despite the abundance of evidence highlighting the significance of policies focused on decreasing the number of expected confirmed cases, political debate has been greatly influenced by the negative impact on economics. Therefore, the debate has directed focus to the possible inefficiencies of such measures and the estimation of an agreeable compromise between protecting the health of citizens and preventing economic damage. Thus, one study determined the effect of lockdown measures on the number of new confirmed cases. Researchers have obtained a greater understanding of this relationship through quantitative panel analysis, the formation of a national and international longitudinal dataset, and estimation of lockdown impact through statistical models. The results of this study support the claim that lockdown is effective in the reduction of new cases in countries that implement such measures versus countries that do not implement these measures [8].

## Review

Discussion on implementation of safety recommendations

The differences in the guidelines can be correlated with the reason why some countries and states have seen lower COVID-19 cases and death rates. Worldwide, countries began taking various precautions based on the recommended guidelines in that specific country, but not all countries were as effective as others. For example, some countries took very effective measures, such as countries in Asia, which have had some experience in dealing with pandemics as recently as 2015, South Korea was hit by an epidemic called middle east respiratory syndrome (MERS) caused by a coronavirus, in 2015 [9]. Therefore, they had something to base their guidelines on, which is why they had more specific recommendations and procedures that targeted the particular problems COVID-19 brought. Additionally, an example of strong implementation of safety recommendations includes China’s police-operated drones encountering people in public, urging them to take better precautions against COVID-19, as shown by videos released by Chinese media outlets even before the pandemic hit the United States [10]. Other countries such as Spain, the United Kingdom, and India initiated nationwide lockdowns to prevent the exponential spread of the virus. All of these different implementations proved somewhat effective due to lower cases, considering just how contagious is COVID-19. America, in comparison, did not implement its recommendations as quickly. For example, 13 states in the U.S. did not require mandatory face masks as of March 2021. Around the same time, Texas joined them by lifting their mask mandate [11]. Although masks were recommended, the slow implementation likely played a part in the rise of cases seen in the U.S. Other deviations involved how schools around the U.S. varied in their implementation processes as well. Many schools required masks but did not reinforce the social distancing recommendation well, which is just as important as wearing masks. Additionally, colleges had different reopening plans back in August 2020 that involved different learning styles as well as testing requirements for those living on-campus. Colleges that did not have aggressive testing vastly impacted the number of cases in their surrounding community [12]. Throughout 2020, New Zealand was held up as one of a few countries that had successfully managed to contain COVID-19. It is widely accepted that this achievement stemmed in large part from the government's decision to close the country's borders and to impose strict lockdowns on its residents to suppress community transmission. Although politically risky and dramatically different from the response taken by most high-income countries, what became known as New Zealand's "elimination" strategy, or "zero-COVID" approach, was increasingly accepted by most New Zealanders as necessary. Overall, implementation of good testing requirements and other safety precautions such as masks and social distancing are key when it comes down to fighting this virus globally. 

Discussion on treatment approaches

Supportive care therapy is centered around treating the symptoms of coronavirus to help prevent the disease from progressing to a more serious state. This type of therapy includes, for example, staying hydrated, social distancing, and monitoring vitals at home for people with mild symptoms. For more serious cases, measures include supplemental oxygen and mechanical ventilatory support, which may be needed for more severe cases [13]. Furthermore, since the beginning of the pandemic, various drugs have been examined for the potential treatment of COVID-19. In March 2020, the U.S. Food and Drug Association (FDA) issued an emergency use authorization (EUA) for the use of hydroxychloroquine (HCQ) and chloroquine (CQ), which are antimalarial agents that are also often used to treat certain autoimmune disorders. At the start of the pandemic, both agents were found to have in vitro activity against SARS-CoV-2, and due to this potential biological finding, various clinical studies were initiated to study the efficacy of these agents for the treatment of COVID-19 [14]. In April 2020, the use of these drugs was advised to only be used in a hospital setting or during clinical trials due to data suggesting that the use of HCQ may be associated with significant cardiac side effects, including the risk of heart rhythm problems in patients with COVID-19. In June 2020, the FDA revoked the EUA after clinical trials showed no evidence that HCQ and CQ could prevent deaths or help the health of COVID-19 positive patients improve faster. Additionally, a drug that was also under examination at the same time was remdesivir which is an antiviral drug that was originally established during the Ebola epidemic in 2013. Remdesivir is the first drug found to have a positive effect on hospitalized patients with COVID-19. Therefore, in May 2020, the FDA approved the use of remdesivir for the treatment of COVID-19 in certain circumstances. Remdesivir shows an antiviral effect on the cells infected with SARS-CoV-2 in both in-vitro and in-vivo studies. When metabolized to its active form, this drug interferes with RNA polymerase and potentially leads to the premature termination of RNA transcription. Continuing research shows that remdesivir reduces the time it takes a patient to clinically improve when the drug is administered early enough in the course of illness of patients with less severe symptoms; however, it does not show an effect on mortality [15]. In addition to supportive care therapy and antiviral drugs, convalescent plasma transfusion and monoclonal antibody treatments have allowed researchers to make enhancements and progress in their efforts to find effective treatment options for COVID-19. Although the primary protective mechanism for both of these immunotherapy treatments is the neutralization of pathogens, the action of monoclonal antibody treatments is more targeted and specific. These two immunotherapy treatments can particularly be useful to the elderly and people with co-morbidities such as cardiovascular disease, lung disease, and diabetes, who are at a higher risk of disease progression and severe symptoms. Along with the previously mentioned treatment approaches, in December 2020, the FDA issued an EUA for the Pfizer-BionTech and Moderna COVID-19 vaccine, and in February 2021, the FDA issued an EUA for the Janssen COVID-19 vaccine. According to The Centers for Disease Control and Prevention, about 101.8 million people have received at least one dose of a COVID-19 vaccine, and on average, about 2.99 million doses are being administered per day [16]. Although there has been a high volume of administration of the vaccine, there have been certain hurdles in the rollout of the vaccine, including limited and uncertain supply of the vaccine, individual states having to make their own decisions on how to administer and distribute vaccines, and the public showing hesitancy for obtaining the vaccine. At the onset of the vaccine distribution, federal and state health officials agreed that early distribution of the vaccine should first target high-risk frontline health care workers and staff at long-term care communities. However, researchers found that these two high-priority groups were among the most reluctant to receive the COVID-19 vaccine [17]. The combination of the vaccine supply is limited, and the hesitancy of the groups who had vaccines reserved for them can cause further delays in the distribution of the vaccine. Also, after the government organized the rapid development of the COVID-19 vaccine, states were left with the responsibility to make their own decisions on how to distribute the vaccine. This has created an inconsistency in the rollout of the vaccine among states due to different plans and urgencies in the administration and distribution of the vaccine. Overall, even though the COVID-19 vaccine is being given to a higher volume of people than expected, the distribution aspect of the vaccine has faced some challenges. 

Approach to the economic shutdown

The World Health Organization (WHO) characterized COVID-19 as a pandemic on March 11, 2020. This virus has caused billions of infections and millions of deaths spanning roughly 213 countries and territories. Not only identified as a public health crisis, but the infection has also severely affected the global economy. The notable economic impact has occurred globally due to loss of life, trade disruption, reduced productivity, and the near extinction of the tourism industry. Serving as a potential “wake-up” call, global leaders can increase cooperation on viral outbreak preparedness and provide financial aid for collective international action. Despite sufficient evidence on the expected health and economic costs of viral outbreaks, numerous countries have failed to appropriately invest in preparedness and preventative measures to reduce the risks of large infectious disease outbreaks [18]. Service industries such as transportation, tourism, and hospitality have faced major losses due to reductions in travel. As a result of quarantine measures, entertainment, travel and transportation, and restaurants and bars are among several sectors to be the worst affected. Global financial markets have also been massively impacted by the effects of the COVID-19 outbreak. The COVID-19 pandemic may also cause a severe impact on the labor market, especially for areas dependent on migration. Globally providing balance in both high- and low-skilled professions, migrant workers provide crucial contributions to labor markets. As quarantine and international travel restrictions are likely to persist in order to decrease and diminish the spread of COVID-19, migration will be severely limited, leading to a hindrance in global economic growth and development. To “flatten the curve,” governments have employed strict measures such as border shutdowns, quarantine, and travel restrictions in areas that compose the world’s largest economies, igniting fear of an imminent economic recession and crisis [19]. To fully understand the turmoil, one study summarized the effects of COVID-19 on several individual aspects of the global economy, providing an extensive focus on primary sectors, industries involved in the extraction of raw materials, secondary sectors, industries involved in the production of competing products, and tertiary sectors, industries involved in providing various services. Oil prices dropped suddenly in one day since the Organization of the Petroleum Exporting Countries (OPEC) refused to decrease oil production. Concerning secondary sectors, the manufacturing industry face and view staffing shortages and importation issues as primary concerns for businesses due to supply chain disruptions and public health policies. As numerous countries are adopting protective measures and due to the nature of supply chains (global overlap), it is expected that these concerns will grow beyond borders, Regarding the numerous industries constituting the tertiary sector, the COVID-19 pandemic has affected all levels of education, ranging from primary to tertiary education, between high-income populations versus low-income populations in terms of technological access for digital learning [20]. In terms of the economic impact, a study conducted by the Brookings Institution modeled closures nationwide and within major U.S. cities and suggested, based upon the results, that there would be an average cost of $142 per learner per week. These findings led to an estimation that a four-week closure within New York City would ensue in an economic cost of $1.1 billion, and a nationwide closure for a 12-weeks period would amount to 1% of GDP [21]. Additionally, the study determined the influence of closures on healthcare workers’ children and calculated that 6-19% of workforce (healthcare) hours have been lost. As COVID-19 has globally affected communities, organizations, and businesses, the global economy, and financial markets have been unintentionally affected. Inefficient governmental responses and policies have caused disruptions within supply chains. Along with these disruptions, the capital market sector has experienced drastic fluctuations in stock value worldwide. This decline in global markets has generated a tense environment with dire liquidity levels. To extinguish these effects, central banks have interceded to make sure liquidity is maintained and to alleviate economic shock. Considered to be the most severely impacted, the hospitality and travel industry has numerous hourly workers who are in danger of facing potentially difficult hardships. Along with the hospitality and travel industry, the tourism sector is presently one of the most impacted industries by the COVID-19 outbreak. The World Travel and Tourism Council has estimated that around 50 million jobs in the tourism and global travel sector may be at risk. Within the United States, the implementation of safety measures such as restriction of non-essential travel, suspension of visa services, and border closures may hasten the disruption of the U.S. economy [22].

## Conclusions

In general, just following the recommendations do not seem sufficient in overcoming this pandemic. There is a good purpose and bias for taking these precautions, but it is how the recommendations are implemented that matter most as the pandemic has been a long battle with no end in sight. While the severity of the global pandemic led to an urgent necessity for effective therapy, there is still a need for high-quality randomized clinical trials to be conducted in order to make definite conclusions about how successful and sufficient a treatment approach is. This study could help the approach of the future pandemic. Although the COVID-19 vaccines have been the most promising in creating a long-term approach, there have been complications in the rollout of the vaccine that will also take further interventions to improve. As the COVID-19 pandemic remains to disrupt economic activity and negatively impact service and manufacturing industries, it is expected that financial markets will continue to be strained. It is still unknown as to whether this ongoing crisis will have a long-term structural influence on the global economy or substantially short-term economic and financial consequences. In either situation, it is clearly evident that diseases such as COVID-19 have the ability to impose severe financial and economic costs on various economic scales. Due to globalization, higher volumes of transportation connectivity, and economic interconnectedness/interdependence, it has been costly and difficult to contain the spread of the virus and to reduce the risks once the virus has spread to several locations. As outbreaks of novel infectious diseases are not expected to disappear within the near future, proactive, collaborative international action is necessary not only to save lives but also to secure economic welfare. In order to be prepared for the next pandemic, prevention, planning, and recognition of the crisis as well as containment are key in order to return the society to its normal pre-pandemic state. Certainly, there are a lot of lessons that can be learned in how this pandemic progressed. 
